# Hypoxia-inducible factor-1: A new neuroprotective agent for the treatment of hyperglycemia after stroke

**DOI:** 10.4103/NRR.NRR-D-25-00794

**Published:** 2026-01-02

**Authors:** María Isabel Hernández, José L. Zugaza, Abraham Martín

**Affiliations:** 1 Achucarro Basque Center for Neuroscience, Leioa, Spain; 2 Department of Genetics, Physical Anthropology and Animal Physiology, Faculty of Science and Technology, EHU, Spain; 3 Ikerbasque Basque Foundation for Science, Bilbao, Spain

**Keywords:** acute hyperglycemia, hypoxia-inducible factor-1α, ischemic stroke, neuroprotective agents, post-translational modifications

## Abstract

Acute hyperglycemia in ischemic stroke occurs in almost half of cases, inducing a worsening of the underlying pathophysiology. Hence, the management of acute hyperglycemia during the first hours following ischemic stroke requires well-defined strategies. Furthermore, the effect of hyperglycemia on stroke remains an unresolved paradoxical process whose possible causes are still unknown. The ischemic process involves the activation of multiple signaling pathways related to cell death and survival, which can be acutely altered by hyperglycemia, causing subsequent worsening of the ischemic process. In search of potential biological targets to combat this medical burden, the role of hypoxia-inducible factor appears as a novel neuroprotective agent that needs further investigation. Furthermore, hypoxia-inducible factor protein targets multiple pathways changing depending on factors such as the severity of hypoxia, the time elapsed after reperfusion and the specific cell type. In fact, the poor prognosis of ischemic stroke associated with acute hyperglycemia has been linked to alterations in hypoxia-inducible factor-1α protein function. Together, these findings support this transcription factor as a new therapeutic target to prevent the negative effect of hyperglycemia, opening new avenues for treatment in stroke.

## Introduction

Stroke is a neurological condition of vascular origin that commonly results from a sudden arterial obstruction, leading to ischemia. In other cases, it stems from bleeding within the brain parenchyma caused by the rupture of a cerebral blood vessel, known as a hemorrhagic stroke (Kuriakose and Xiao, 2020; Hilkens et al., 2024). In ischemic stroke, a reduction in cerebral blood flow of more than 50% leads to reversible neuronal dysfunction, and if the ischemia persists for long enough, irreversible tissue injury takes place. The time from the onset of symptoms to irreversible tissue injury depends on the magnitude and duration of the reduction in cerebral blood flow (Feske, 2021). Globally, stroke represents one of the most significant contributors to both mortality and persistent functional impairment (Katan and Luft, 2018, 2021). In early 2000s Europe, about 1.1 million individuals suffered a stroke each year, with case-fatality rates within the first month ranging from 13% to 35% (Chamorro et al., 2016). Additionally, the aging population trend suggests a continuous increase in these statistics. Therefore, stroke is undeniably an emergency and a critical issue for researchers in both basic science and clinical neuroscience.

In ischemic stroke, intravenous administration of recombinant tissue plasminogen activator (tPA) administered within a few hours of symptom onset significantly increases the likelihood of patients experiencing minimal or no disability at 90 days (at least 30% more likely). However, thrombolysis with tPA is largely limited to a time window of 4.5 hours after symptom onset. Together with endovascular thrombectomy, tPA is one of only Food and Drug Administration-approved therapies that has been shown to be effective in reducing brain infarct volume (Herpich and Rincon, 2020; Hilkens et al., 2024). However, several studies have shown that acute hyperglycemia reduces the efficacy of tPA as well as the outcome of stroke (Pandolfi et al., 2001; Salman et al., 2022).

The presence of acute hyperglycemia during ischemic stroke has been described during the first hours (Christensen and Boysen, 2002). Although diabetes is considered a risk factor for stroke (Bradley et al., 2022), the mechanisms that trigger acute hyperglycemia in the brain remain controversial. Patients lacking a diabetes diagnosis tend to experience particularly unfavorable results, and initiating insulin therapy within 24 hours has not shown a significant advantage (Capes et al., 2001; McCormick et al., 2008). In several preclinical studies, predominantly in rodents, hyperglycemia during ischemic stroke appears to exacerbate lactic acidosis, oxidative stress, inflammation, and blood–brain barrier (BBB) permeability, further worsening the hypoxic brain injury (Lin et al., 1998; MacDougall and Muir, 2011; Ferrari et al., 2022).

In hypoxic conditions, cells rapidly adapt to maintain energy demands through hypoxia-inducible factor (HIF), a mechanism conserved across different cell types. Under normal oxygen conditions or normoxia, HIF-1α subunit-protein undergoes degradation. However, under hypoxic conditions, the degradation of the HIF-1α subunit is inhibited, allowing it to translocate to the nucleus. There, it associates with the β-subunit and other cofactors to play as a transcription factor, altering the cellular genetic program (Kewley et al., 2004). During an acute stroke, HIF acts rapidly, although it has been attributed both protective and detrimental functions (Davis et al., 2019; Mitroshina et al., 2021). Moreover, recent molecular studies indicate that the stability of HIF-1α protein is regulated by multiple factors and its post-translational modifications, not solely by oxygen levels but also by hyperglycemic conditions (Ke and Costa, 2006; Kuschel et al., 2012; Albanese et al., 2021). Acute hyperglycemia in the first hours of ischemic stroke has been shown to trigger alterations in HIF-1α protein expression (Bullock et al., 2009; Chen et al., 2010). However, the mechanism behind the negative effect of acute hyperglycemia in stroke remains a challenge, particularly in understanding its role in HIF-1α degradation or activation that may affect the genetic program and the progression of the pathology.

## Search Strategy

For this narrative review, a comprehensive literature search was performed using the PubMed database. Search terms included: MCAO, hyperglycemia, ischemic stroke, HIF-1 alpha, hypoxia, post-translational modifications, and high glucose, which were combined using Boolean operators. Additional focused terms such as succinylation of HIF-1, drugs for HIF-1 alpha, roxadustat in cerebral ischemia, and inhibitors for prolyl hydroxylase (PHD) were also explored. No restrictions were applied regarding publication date, language, article type, or other parameters. The search was conducted between July 2024 and September 2025.

## Hyperglycemia in Stroke

Not only oxygen deprivation, but also glucose deficiency, contributes to ischemic brain damage (Robbins and Swanson, 2014). Despite expectations, acute hyperglycemia occurring in stroke patients is correlated with a more unfavorable prognosis (Yao et al., 2023). In a systematic analysis of observational research on hyperglycemia’s prognostic value in acute stroke, non-diabetic individuals were found to have an increased relative risk of mortality within 30 days of hospital admission (Capes et al., 2001). Blood glucose levels range from 4.4 and 6.1 mmol/L (79–110 mg/dL) in both rodents and humans. Across various stroke researches, the definition of hyperglycemia varies, with cut-off values for glucose levels ranging between 6.1 and greater than 10 mmol/L (110–180 mg/dL). Using these criteria, acute hyperglycemia is present in 30% to 60% of stroke patients (Capes et al., 2001; Bruno et al., 2008; Robbins and Swanson, 2014).

Stress-related hyperglycemia is a physiological reaction resembling the activation of the sympathetic nervous system, primarily driven by the release of stress hormones and pro-inflammatory cytokines in response to tissue damage. This transient rise in blood glucose results from a short-term elevation in plasma glucose levels, reduced insulin production, and increased insulin resistance in peripheral tissues (Bosarge and Kerby, 2013). An essential component of this response involves activation of the hypothalamic–pituitary–adrenal axis, resulting in elevated cortisol concentrations in circulation. Cortisol increases glucose availability by activating key enzymes responsible for gluconeogenesis in the liver and by suppressing glucose uptake in peripheral tissues. Furthermore, cortisol, along with tumor necrosis factor alpha (TNF-α) produced by local tissues, can stimulate glucagon release. Hormones such as catecholamines, cortisol, and glucagon regulate blood glucose levels by promoting gluconeogenesis and glycogenolysis in the liver. This state typically persists until the inflammatory mediators decrease and the catabolic state resolves (Bosarge and Kerby, 2013; Marik and Bellomo, 2013; Zhang et al., 2014; Yao et al., 2023).

Maintaining blood glucose below 110 mg/dL through aggressive insulin treatment has been proven to markedly decrease mortality rates in intensive care units, as well as overall hospital mortality and complications in critically ill individuals (van den Berghe et al., 2001). However, there is ongoing debate regarding the optimal blood glucose to target (Vanhorebeek et al., 2018).

It is hypothesized that insulin exerts neuroprotection by interacting with insulin-like growth factor (IGF) receptors. This hormone promotes nitric oxide (NO) synthesis, which in turn reduces endothelial cell adhesion molecules and proinflammatory cytokines, thereby facilitating vasodilation (McCormick et al., 2008). Bruno et al. (2008) demonstrated the effectiveness of hyperglycemia correction in patients with diabetes mellitus using an intravenous insulin protocol within the first 12 hours following acute cerebral infarction, which did not result in a better prognosis at 3 months. In non-diabetics, it has been observed that during the first 12 hours after a stroke, blood glucose levels fluctuate, and greater hyperglycemia is associated with a worse short-term prognosis, although it does not determine the outcome at 3 months (Christensen and Boysen, 2002). A controversy exists regarding the method of insulin administration in non-diabetic patients during acute stroke (Johnston et al., 2019; Torbey et al., 2022). Despite this, it is generally concluded that hyperglycemia must be controlled, but taking into account avoiding hypoglycemia (Fuentes et al., 2018).

**Figure 1:**
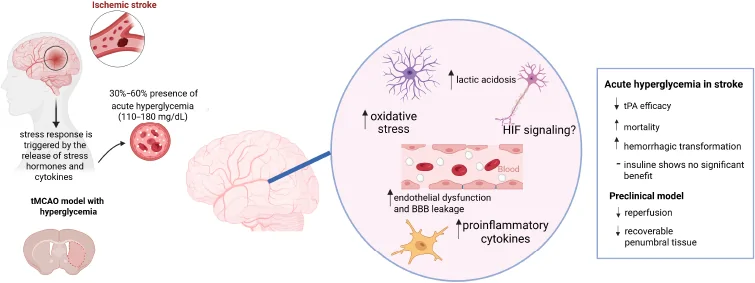
Schematic representation of the deleterious effects of acute hyperglycemia in clinical and preclinical ischemic stroke. Several mechanisms and their consequences are illustrated. Created with BioRender.com. BBB: Blood–brain barrier; HIF: hypoxia-inducible factor; tMCAO: transient middle cerebral artery occlusion; tPA: tissue plasminogen activator.

The overall underwhelming results from insulin clinical studies, including occurrences of hypoglycemia, emphasize the importance of developing more targeted treatment strategies. Moreover, elevated blood glucose levels during acute phases have been associated with an increased risk of hemorrhagic transformation (Mi et al., 2018). Serum glucose concentration showed a positive association with hemorrhagic transformation incidence, as 25% of patients treated with tPA and serum glucose levels over 200 mg/dL experienced symptomatic hemorrhage, whereas this rate was 5% in patients with glucose levels under 100 mg/dL (Demchuk et al., 1999). Both elevated blood glucose and high insulin levels raise plasminogen activator inhibitor concentrations and notably decrease tPA levels, thus impacting thrombolytic treatment (Ferrari et al., 2022; **Figure 1**).

### Effects of acute hyperglycemia in preclinical ischemic stroke

Although several studies have been conducted in patients, animal models remain the best tool for studying in detail how hyperglycemia can exacerbate hypoxic damage (Ferrari et al., 2022), because human studies cannot distinguish cause and effect owing to the lack of control over the extent of the primary ischemic damage. Nevertheless, Parsons et al. (2002) analyzed how acute hyperglycemia in patients with transient ischemia is linked to less recoverable penumbra tissue, concluding, as many other authors have, that hyperglycemia is particularly detrimental in ischemia/reperfusion injury (Quast et al., 1997; Martini and Kent, 2007).

High blood glucose after the onset of acute stroke and the associated increased damage have been predominantly described in rodents (Lin et al., 1998; Martín et al., 2006; Huang et al., 2019; Ferrari et al., 2022). The transient middle cerebral artery occlusion (tMCAO) model in rats and mice has been shown to induce both cell death and survival pathways. The presence of oxidative stress, apoptosis, inflammation, and subsequently angiogenesis in the tissue have been described depending on the region and over time (Krafft et al., 2012; Kumar et al., 2016; Li and Zhang, 2021; Ferrari et al., 2022).

Regarding some of the worsening mechanisms induced by hyperglycemia under cerebral ischemia, more lactic acidosis and oxidative stress have been included, as well as an increasing permeability of the BBB and inflammation (Lin et al., 2000; Kamada et al., 2007; Robbins and Swanson, 2014; Huang et al., 2019; **Figure 1**). Likewise, hyperglycemia induced by streptozotocin results in a significantly greater expansion of infarct volumes than that caused by dextrose infusion, and a review of several studies showed that insulin use did not change the outcome (MacDougall and Muir, 2011). A study by Kamada et al. (2007) demonstrated increased oxidative stress when hyperglycemia was induced three days prior to ischemia. Changes in O-glycosylated proteins were also observed in hyperglycemic rats with hyperglycemia induced 30 minutes before tMCAO (Martín et al., 2006). Vascular permeability increases due to endothelial cell dysfunction particularly after recanalization or reperfusion (Quast et al., 1997; Martini and Kent, 2007). In the context of hyperglycemia, endothelial impairment may result from the enhanced expression of cellular adhesion molecules and the glycation of essential proteins responsible for generating vasodilators and antithrombotic factors, including NO (Amore et al., 1997).

Moreover, elevated glucose levels activate the lipoxygenase and cyclooxygenase pathways within vascular smooth muscle, resulting in increased production of vasoconstrictive prostaglandins such as thromboxane A₂ (Natarajan et al., 1993; Amore et al., 1997). Hyperglycemia can also exacerbate reperfusion injury by providing an increased glucose substrate for superoxide production via nicotinamide adenine dinucleotide phosphate (NADPH) oxidase 2 in different types of cells (Brennan-Minnella et al., 2015). The signaling cascade involving phosphatidylinositol 3-kinase (PI3K), protein kinase B (Akt), and mammalian target of rapamycin, along with the Akt-dependent regulation of nuclear factor kappa-light-chain-enhancer of activated B cells (NF-κB) activation, is recognized for its role in various pathological conditions. Elevated glucose levels stimulate reactive oxygen species (ROS) production, which functions as a critical signaling mediator in neuronal activation, triggering inflammatory and neurodegenerative responses through Akt and extracellular signal-regulated kinases 1 and 2 pathways (Kumar et al., 2016). Furthermore, cerebral mitogen-activated protein kinases (MAPKs) may affect the balance between proteins that promote cell death and those that support cell survival. Downregulation of MAPKs is related to an increase in glucose transporter (GLUTs) content, which is beneficial following stroke. GLUT expression is also modulated by HIF, which is dependent on the redox status (Zhang et al., 2014; Sharma et al., 2022; González et al., 2023). Therefore, acute hyperglycemia could be changing different pathways at the cellular level, but the specific ones remain not fully understood.

## Role of Hypoxia-Inducible Factor in Ischemia

### General mechanism of hypoxia-inducible factor degradation under normoxia

Transcription factors include proteins characterized by the presence of basic helix-loop-helix (bHLH) and Per-Arnt-Sim (PAS) motifs. Mammalian members of the bHLH-PAS family are key regulators of gene expression networks that support numerous critical developmental, physiological, and disease-related processes. bHLH–PAS proteins contain several conserved functional domains: a bHLH domain at the N-terminus that facilitates DNA binding, two tandem PAS domains (PAS-A and PAS-B), and a domain at the C-terminus that activates or inhibits transcription of target genes (Kewley et al., 2004; Edwards and Gorelick, 2022). The transcriptional reactions to environmental pollutants and reduced oxygen levels are mediated by the aryl hydrocarbon receptor and HIF, respectively, while neural development is regulated by the single-minded protein family. Hypoxic responses are regulated by three bHLH-PAS proteins: HIF-1α, HIF-2α (also known as Endothelial PAS domain protein 1, HIF-like factor, and member of the PAS family 2), and HIF-3α (Wang et al., 1995; Kewley et al., 2004; Ke and Costa, 2006).

HIF-1 is a heterodimeric transcription factor consisting of a 120-kDa HIF α-subunit complexed with a 91- to 94-kDa HIF β-subunit (ARNT) isoform. Various competing members of the bHLH-PAS family, such as ARNT and its different variants, are co-expressed and show a range of dimerization activities, which implies the existence of complex regulatory systems governing the interaction of signaling pathways and the precision of gene expression regulation (Kewley et al., 2004).

ARNT encodes a series of common subunits utilized by both HIF-1 and aryl hydrocarbon receptor, depending on the specificity conferred by their PAS homology domains. This process is analogous to the heterodimerization of E2A gene products with various bHLH proteins, which also function as transcription factors. In normoxia, the HIF-1α subunit is located in the cytoplasm, where it is degraded, while the β-subunit is constitutively expressed and localized within the nucleus. In response to hypoxic conditions, the dimer formed by hypoxia-inducible factor-1 alpha and beta (HIF-1α/β) modulates gene expression through binding to a core DNA motif (G/ACGTG) located in hypoxia-response elements. The body’s systemic, localized, and cellular responses to hypoxia, a state characterized by insufficient oxygen supply relative to demand, trigger adaptations that increase oxygen availability or shift metabolism to pathways that do not rely on oxygen (Kewley et al., 2004).

HIF-1α and HIF-2α share 48% amino acid sequence identity and bind to similar promoter sites but differ in the cofactors they recruit and their functions. While the two proteins largely overlapped in their roles, some non-redundant functions have been described depending on the cell type. HIF-3α was discovered later, plays a minor role, and may regulate other HIF isoforms (Kewley et al., 2004; Chen et al., 2018). HIF-1α shows ubiquitous expression, whereas HIF-2α is predominantly present in vascular endothelial cells during the embryonic stage. For example, HIF-1α stimulates glucose metabolism in cells, a function that HIF-2α does not share (Hu et al., 2003). Under cultured conditions, Western blot analysis revealed that both HIF-1α and HIF-2α proteins are present in cortical neurons and astrocytes, although their relative abundance differs. HIF-1α expression is more prominent in neurons, whereas HIF-2α levels are higher in astrocytes (Chavez et al., 2006). Both the severity of hypoxic conditions and glucose levels seem to modulate the relative activities of HIF-1 and HIF-2 in astrocytes, which leads to diverse effects on neurons (Guo et al., 2019).

**Figure 2:**
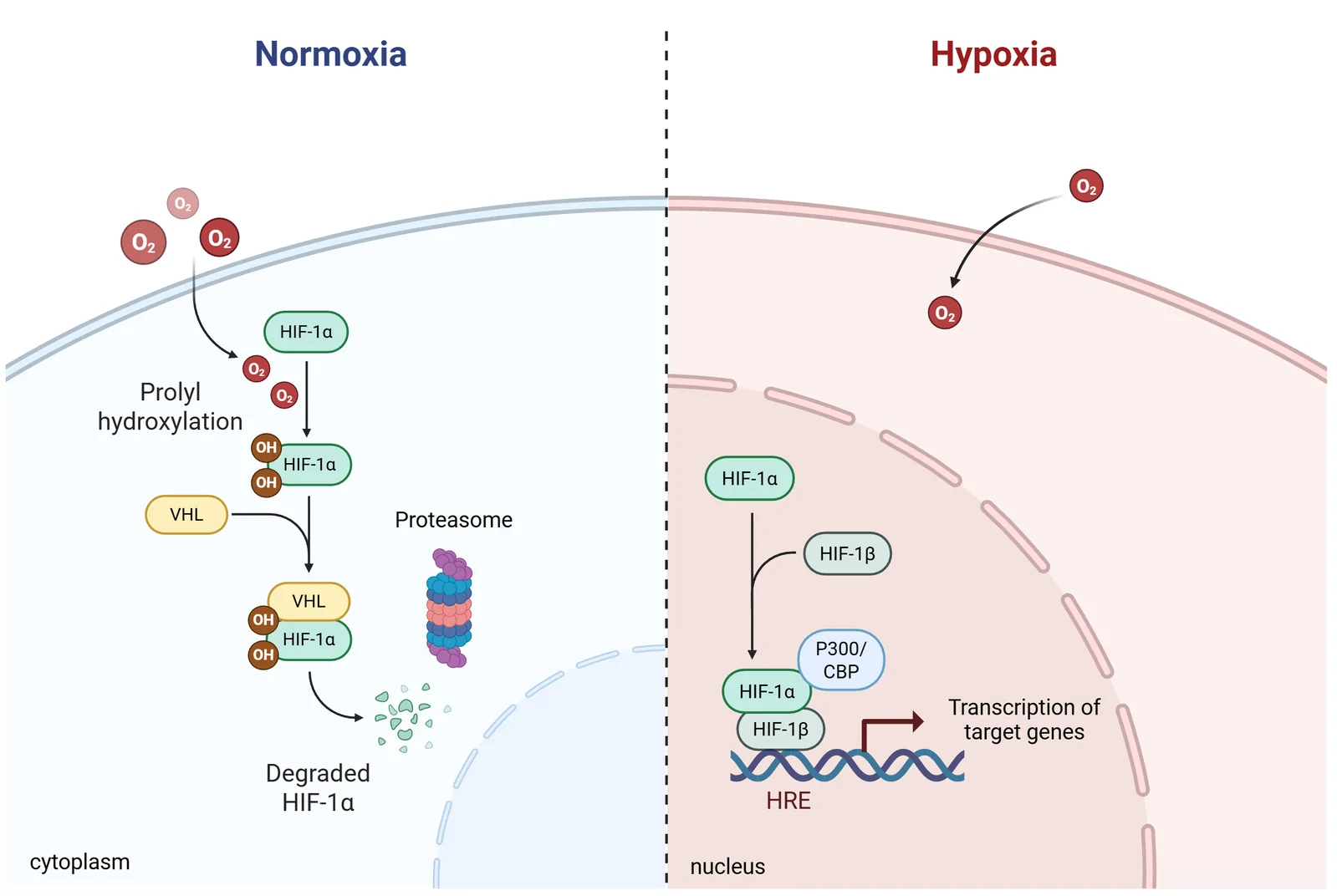
Regulation of hypoxia-inducible factor (HIF)-1α during normoxia and hypoxia. Under normoxic conditions, HIF-1α is hydroxylated by prolyl hydroxylase domain enzymes in the presence of iron (Fe²⁺). This hydroxylation promotes the recruitment of the von Hippel-Lindau protein (pVHL), which acts as a recognition component of the ubiquitin ligase complex. This process leads to the ubiquitination and subsequent proteasomal degradation of HIF-1α. Under hypoxic conditions, hydroxylation is inhibited, resulting in the stabilization of HIF-1α. Stabilized HIF-1α translocates to the nucleus, where it heterodimerizes with the constitutively expressed HIF-1β. This HIF-1α/β dimer then binds to hypoxia-responsive elements (HREs) in the promoter regions of target genes and interacts with transcriptional co-activators p300 and CREB-binding protein (p300/CBP), thereby activating the transcription of HIF-1α target genes. Created with BioRender.com.

Although the function varies between isoforms, the mechanism of HIF α-subunit degradation in normoxia is the same. HIF-1α is continuously synthesized and rapidly degraded by the ubiquitin-proteasome system (Huang et al., 1998). The enzyme PHD, which requires oxygen and iron, catalyzes the hydroxylation of HIF-1α at two proline sites (P564 and P402 for human HIF-1α, and P530 and P405 for human HIF-2α) located in the oxygen-dependent degradation domain. These hydroxylated prolines act as binding sites for the von Hippel–Lindau tumor suppressor protein (pVHL), which associates with Elongin B and C, Cullin 2, and RING-box protein 1 to form a functional E3 ubiquitin-protein ligase complex. This complex mediates the ubiquitination of HIF-1α, directing it towards degradation (Kewley et al., 2004; **Figure 2**).

During normoxia, a second hydroxylation event is mediated by the factor inhibiting HIF-1 (FIH-1), which is also known as asparaginyl hydroxylase. An asparagine residue (N803 in HIF-1α and N851 in HIF-2α), located within the C-terminal transactivation domain, is hydroxylated, preventing the recruitment of the co-activator p300 and CREB-binding protein (p300/CBP) and the subsequent transactivation of target genes. During hypoxia, the decreased oxygen concentration inactivates PHD and FIH-1, leading to the buildup of HIF-1α. HIF-1α then dimerizes with its β-subunit in the nucleus, recognizes hypoxia-response elements present in the enhancer regions of multiple genes, associates with p300/CBP, and triggers gene transcription (Kewley et al., 2004). Taking this into account, the function of HIF depends on the α-subunit. The ability of HIF-1α to regulate transcription depends on its nuclear levels and its interaction with the HIF β-subunit to form a heterodimer that binds DNA (Mottet et al., 2003; Flügel et al., 2007; Kalousi et al., 2010).

Tuomisto et al. (2004) showed significant induction of the hypoxia-inducible angiogenic pathway involving HIF-1α and HIF-2α in acute-on-chronic ischemia in muscle using DNA array. Vascular endothelial growth factor (VEGF), vascular endothelial growth factor receptor 2 (VEGFR-2), as well as TNF-α and its downstream signaling machinery promoting inflammation and cell death, were also upregulated. In the chronic situation, the anabolic and survival factors IGF-1 and IGF-2 were upregulated in atrophic and regenerating myocytes, along with attenuated expression of HIF, VEGF, and VEGFR-2 in the same cells (Tuomisto et al., 2004).

The pioneering study by Semenza et al. (1997), along with recent research on the structure and function of HIF-1, has significantly advanced our understanding of its role during ischemia (Semenza et al., 1997). Current studies suggest that HIF-1 may also be regulated by factors such as mitochondria-derived ROS. However, the idea that ROS regulates HIF stabilization or degradation remains controversial, highlighting the variability of this transcription factor’s behavior depending on cell type and hypoxic conditions (Semenza, 2001; Shi, 2009; Hagen, 2012; Chen et al., 2018; Taylor and Scholz, 2022). Evidence suggests that the cellular response to hypoxia — whether adaptation, injury, or cell death — is influenced by both HIF signaling pathways and the unique signaling environments of different cell types (Semenza, 2001).

**Effects of hypoxia-inducible factor 1α in stroke**

Preventing neuronal death and enhancing recovery after ischemic injury remain key goals in stroke therapeutics. Over recent decades, extensive research on hypoxia adaptation mechanisms-particularly the functions of HIF- culminated in the 2019 Nobel Prize in Physiology or Medicine, which was jointly awarded to William G. Kaelin Jr, Sir Peter J. Ratcliffe, and Gregg L. Semenza “for their discoveries of how cells sense and adapt to oxygen availability.” While HIFs are gaining attention as potential therapeutic targets for hypoxia-related diseases, their role in cerebral ischemia remains unclear, and the appropriateness of HIF stabilization as a strategy is still under debate (Davis et al., 2019; Mitroshina et al., 2021).

A crucial objective is to develop neuroprotective methods that prevent injury to brain cells from ischemia and reperfusion, while also expanding the effective time window for administering thrombolytic treatment. Previous studies have frequently tested neuroprotective agents in young animals, which do not fully replicate stroke in older humans. In the current era of thrombectomy, combining neuroprotective agents with recanalization therapies may improve the delivery of these agents to specific brain regions, thereby enhancing the efficacy of stroke treatments (Feske, 2021; Paul and Candelario-Jalil, 2021).

#### Pathophysiology of stroke

The progression of ischemic brain damage following impaired blood flow begins with the formation of a core of irreversibly injured necrotic tissue within the affected region, followed by the development of late-phase injury in the peri-infarct area, a potentially salvageable region surrounding the core (Ratan et al., 2007; Broughton et al., 2009). Neuronal damage is associated with excitotoxicity in the penumbral zone during ischemia/reperfusion. When brain tissue is deprived of oxygen and glucose, energy-dependent ion pumps and channels fail immediately, resulting in the release of harmful concentrations of excitatory neurotransmitters. Excessive glutamate release, an important excitatory neurotransmitter, plays a key role in triggering brain damage. This process includes the activation of N-methyl-D-aspartate receptors, L-type voltage-gated calcium channels, and post-synaptic quisqualate receptor complexes (Paul and Candelario-Jalil, 2021).

The entry of calcium via N-methyl-D-aspartate receptors triggers intracellular signaling cascades such as mitochondrial depolarization, cytosolic phospholipase A2 activation, arachidonic acid metabolism by cyclooxygenase-2, and activation of NADPH oxidase. All of this leads to increased free radical production. Deficient function of glutamate transporters is also correlated with increased lactic acidosis. Excess glucose molecules cannot be metabolized through the aerobic oxidative pathway, and are instead oxidized through anaerobic glycolysis, leading to the production of lactic acid (Camacho and Massieu, 2006).

The resulting oxidative and nitrosative stress is exacerbated during reperfusion, as the restored oxygen supply induces lipid peroxidation, protein oxidation, and DNA damage, ultimately leading to apoptosis. Furthermore, oxidative stress promotes the release of damage-associated molecular patterns, which trigger an inflammatory response. This response involves activation of brain-intrinsic microglia, increased BBB permeability, and infiltration of peripheral immune cells, further contributing to ischemic injury (Camacho and Massieu, 2006; Gülke et al., 2018; Sun et al., 2018; Buscemi et al., 2019; Paul and Candelario-Jalil, 2021).

In the initial phase of stroke, mitochondria are the primary source of ROS. The depletion of adenosine triphosphate leads to elevated calcium concentrations in neurons, triggering a large-scale production of ROS via mitochondrial depolarization. Furthermore, the activation of NADPH oxidase in infiltrating macrophages and other immune cells during neuroinflammatory responses adds to ROS generation, a phenomenon known as the “oxygen burst” that occurs during reperfusion (Sun et al., 2018).

Current treatments for ischemic stroke focus on achieving rapid reperfusion through intravenous thrombolysis and/or endovascular thrombectomy, both of which have been shown to effectively reduce disability. Restoring oxygen and glucose delivery to the ischemic brain through these interventions is essential for minimizing infarct size. Nonetheless, reperfusion introduces peripheral immune cells to the damaged brain tissue, resulting in amplified immune activation and inflammatory injury. Damage-associated molecular patterns stimulate the production of cytokines and chemokines involved in inflammation. Chemokine receptors recruit leukocytes to the brain, exacerbating neurological outcomes. This inflammatory cascade activates microglia and astrocytes, prompting their proliferation and the production of additional cytokines such as interleukin (IL)-1, IL-6, TNF-α, among others. Moreover, proinflammatory cytokines stimulates increased expression of endothelial adhesion molecules, such as selectins and intercellular and vascular adhesion molecules, allowing for the transmigration of peripheral immune cells across the BBB (DeLong et al., 2022).

The sub-acute phase of stroke is characterized by the infiltration of immune cells into the brain parenchyma and abundant secretion of pro-inflammatory cytokines. However, the immune response is highly complex, as evidence suggests that even during the acute phase, it can have beneficial effects that contribute to improved functional outcomes. Alternative immune activation, unlike classical activation, is associated with reduced inflammation, decreased neurological damage, and increased tissue repair (Jayaraj et al., 2019; DeLong et al., 2022; Taylor and Scholz, 2022).

#### Role of hypoxia-inducible factor 1α in stroke

**Figure 3:**
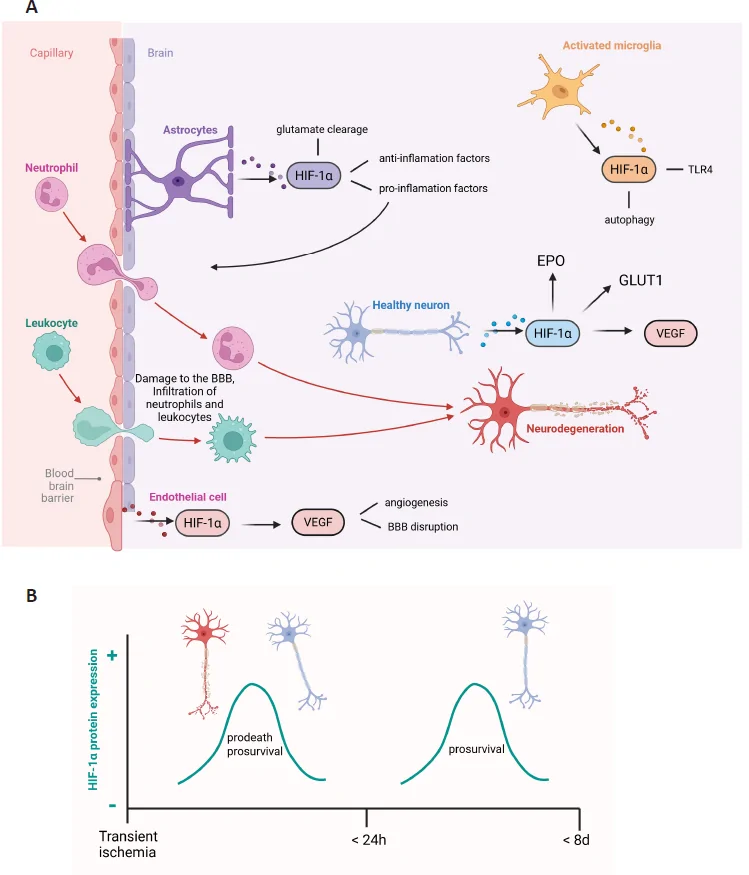
Physiological functions of HIF-1α. (A) HIF-1α and its target genes in various cell types of the neurovascular unit, including neurons, endothelial cells, astrocytes, and microglia, during ischemic stroke. (B) Time-dependent roles of HIF-1α in neurons following transient ischemia. Created with BioRender.com. BBB: Blood–brain barrier; EPO: erythropoietin; GLUT1: glucose transporter 1; HIF-1α: hypoxia-inducible factor 1α; TLR4: toll-like receptor 4; VEGF: vascular endothelial growth factor.

In ischemic stroke, HIF is one of the transcription factors that are activated, but its role remains controversial, as both beneficial and harmful effects have been reported. Recent evidence suggests that this duality is influenced by the severity of ischemia and the type of cell involved (**Figure 3**; Koh et al., 2015; Davis et al., 2019). In the ischemic brain, VEGFs are key regulators of HIF-1α-induced neuroprotection and neurogenesis, along with erythropoietin and GLUT1. Neuroprotective effects of VEGF are believed to be mediated by regulating neurogenesis and angiogenesis in ischemic brain tissue, notably at 12 hours post-ischemia. In the early phase of ischemic stroke, VEGF produced by HIF-1α-activated endothelial cells increases capillary permeability, facilitating endothelial-to-mesenchymal transformation and causing BBB breakdown. Likewise, VEGF also influences ischemic brain tissue by modulating apoptotic factors. In general, HIF-1α activation is considered detrimental immediately after an acute ischemic stroke (e.g., within 1 hour; He et al., 2021; Amin et al., 2024).

Experimental findings suggest that suppressing HIF-1α expression during the initial stages of hypoxia can promote increased cell survival in both *in vitro* and *in vivo* settings. However, in animal models, early HIF-1α expression induces genes that promote glucose metabolism, angiogenesis, erythropoiesis, and cell survival. HIF-1α induces early expression of erythropoietin, attenuating the inflammatory response by decreasing the expression of cyclooxygenase-2 and inducible NO synthase. Besides, HIF-1α is related to the suppression of microglial activation, the inactivation of autophagy, and the expression of anti-apoptotic proteins (Amin et al., 2021; Mitroshina et al., 2021). HIF-1α inhibition could be useful in later stages of hypoxia. In this controversial context, HIF-1α may induce cell death under conditions of severe and prolonged ischemia, yet it promotes cell survival following less intense ischemic injury (Shi, 2009).

In cases of transient cerebral ischemia, particularly with a focus on neurons, the effects of HIF-1α expression appear to be highly dependent on the timing of reperfusion (Baranova et al., 2007; Yeh et al., 2011). Baranova et al. (2007) found that removing HIF-1α specifically in neurons worsened ischemic damage in a mouse model of tMCAO lasting 30 minutes. In genetically engineered mice lacking neuronal HIF-1α, they observed larger infarct areas and decreased survival rates compared to wild-type mice exposed to the same ischemic conditions. Their results indicate that although early HIF-1α expression (within 24 hours after ischemia) may trigger the transcription of pro-apoptotic genes that contribute to neuronal injury, this early harmful phase is eventually surpassed by a prolonged neuroprotective effect mediated by the upregulation of genes involved in tissue repair and cell survival (Baranova et al., 2007).

In contrast, Yeh et al. (2011) employed a rat model of focal ischemia induced by endothelin-1 for one hour and identified a biphasic pattern in HIF-1α expression, with an initial peak occurring between 1 and 12 hours, followed by a second peak at 48 hours post-ischemia. Their research demonstrated that administering 2-methoxyestradiol, a HIF-1α inhibitor, 30 minutes after the ischemic event significantly decreased infarct size, while administration at 8 hours post-ischemia showed no neuroprotective benefit. These findings imply that inhibiting HIF-1α early after ischemia is advantageous, but blocking it later may disrupt repair mechanisms, highlighting the complex and time-dependent functions of HIF-1α in ischemic brain injury (Yeh et al., 2011; **Figure 3**).

Apart from the time after ischemia and severity, cell type is also influencing HIF-1α functions. Regarding the first steps of the pathophysiology of stroke, it is well established that astrocytes can rapidly remove neurotransmitters, such as glutamate, released into the synaptic cleft. Additionally, HIF-1α defends astrocytes against glutamate toxicity caused by hypoxia. During the initial stages of ischemic stroke, astrocytes have been identified as negative modulators of neuroinflammation through secretion of transforming growth factor-β, IL-4, and IL-1. As ischemic injury worsens and neuroinflammation intensifies, astrocytes release HIF-1α-dependent proinflammatory cytokines, including IL-1β, IL-6, and TNF-α. Overactivation of microglia results in excessive, uncontrolled inflammation that can damage tissue and is considered a critical factor in injury during the subacute stage of ischemic stroke. Moreover, targeting HIF-1α in microglia through therapeutic strategies has exhibited neuroprotective effects (He et al., 2021; Amin et al., 2024).

Astrocytes are also known to play a protective role in neurons and are crucial in the defense against oxidative stress (Koh et al., 2015). Vangeison et al. (2008) demonstrated that selective loss of HIF-1α function in astrocytes markedly reduced hypoxia-induced neuronal death in co-cultures, whereas loss of HIF-1α function in neurons increased susceptibility to hypoxia-induced neuronal death (Vangeison et al., 2008). In animal models, Koh et al. (2015) found that activation of hypoxia-induced glial T-cell immunoglobulin and mucin domain-containing protein 3 (TIM-3) by HIF-1/TIM-3 axis in astrocytes promotes the production of neutrophil attractants and the recruitment of neutrophils to hypoxic sites. However, an aberrant increase in neutrophil infiltration leads to abnormal inflammation and subsequent pathological events in the brain (Koh et al., 2015). According to Jolly et al. (2011), hypoxia upregulates retinoid-related orphan receptor-α in astrocytes and neurons; however, retinoid-related orphan receptor-α inhibits HIF-1α expression only in astrocytes. Consequently, HIF-1α might be detrimental to neurons if generated by astrocytes but provides neuroprotection when produced by neurons (Vangeison et al., 2008; Gürer et al., 2009; Jolly et al., 2011; Takahashi, 2021).

Since stroke does not represent a single, homogeneous category of injury, it is not an ideal candidate for a one-size-fits-all treatment approach. Compounds targeting individual components of the ischemic cascade have largely failed to translate into clinical practice for treating ischemic diseases. An alternative strategy is to target entire physiological networks that influence multiple pathways simultaneously, thereby suppressing both ischemic and reperfusion damage. Involving proteins such as HIF-1α, tolerance following preconditioning stimuli unfolds in two stages: an acute phase marked by rapid post-translational modifications, and a subsequent delayed phase lasting from one to three days that is controlled by gene expression changes (Shi, 2009; Heyman et al., 2011; Chen et al., 2018). In mild hypoxia, HIF-1α appears to be beneficial, but more data are needed to clarify its role in neurons and other glial cells. Hence, the role of HIF-1 in the penumbra region, which represents the potentially recoverable area after a stroke, should be studied in greater detail.

#### Does acute hyperglycemia affect hypoxia-inducible factor 1α?

Observations strongly suggest that diabetic complications are, at least in part, related to the loss of the cellular response to hypoxia. In diabetic tissues, the expression of endothelial NO synthase, chemokines CXCL12, CXCR4, VEGF, and other growth factors is decreased. Multiple gene therapy approaches aimed at indirectly boosting VEGF production have been reported to enhance hypoxia-driven wound repair and angiogenic responses in diabetic animals. Destabilization of HIF-1α is likely the key event that transduces hyperglycemia into the loss of cellular responses to hypoxia in many diabetic complications, although the exact mechanism remains unknown (Bento and Pereira, 2011; Catrina, 2014; Bradley et al., 2022). In patients with diabetic hyperglycemia, chronic exposure to elevated glucose levels may lead to adaptive changes at the cellular level, aimed at mitigating the toxic effects of prolonged glucose elevation over time (Bosarge and Kerby, 2013).

Hence, could changes in HIF-1α during ischemic stroke contribute to the worsening effects of acute hyperglycemia? Bullock et al. (2009) demonstrated in a 30-minute murine model of tMCAO with hyperglycemia induced 30 minutes before the occlusion, that preischemic hyperglycemia significantly increased infarct volume in superoxide dismutase 2 (SOD2) deficient mutant (SOD2^–/+^) mice. In this model, HIF-1α protein levels were significantly reduced in the ischemic core at both 5 and 24 hours of reperfusion (Bullock et al., 2009). These findings suggest that the increased brain damage observed in hyperglycemic SOD2^−/+^ mice could be associated with HIF-1α suppression. In a 90-minute rat model of tMCAO with hyperglycemia induced 15 minutes before the procedure, inhibition of HIF-1α and its dependent genes were shown to attenuate hemorrhagic transformation at the reperfusion stage (Chen et al., 2010). However, no data were provided on whether hyperglycemia directly induces increased HIF-1α expression. Moreover, inhibiting HIF-1 activity through specific endothelial HIF-1α deletion alleviated BBB leakage and diminished brain infarction in diabetic animals. Insulin treatment for glycemic regulation also suppressed HIF-1α overexpression, resulting in lower BBB permeability and reduced infarct volume (Zhang et al., 2016). These findings highlight the complexity and model-dependent nature of the role of HIF-1α in ischemic stroke with hyperglycemia, as there is no conclusive data yet on its precise function.

Conversely, very few *in vitro* studies have examined the combined effects of hyperglycemia and hypoxia in neurons or glial cells. More commonly, studies investigate these variables separately, such as those demonstrating the damaging effects of high glucose in astrocytes (Rivera-Aponte et al., 2015; Li et al., 2018). However, research on their combined effects has also emerged. For example, Guo et al. (2019) showed that the severity of hypoxia and the abundance of glucose regulate the balance between HIF-1α and HIF-2α activity in astrocytes, leading to diverse effects on neurons (Guo et al., 2019). Similarly, Orellana et al. (2010) found that high glucose in hypoxia reduces cortical astrocyte viability.

**Figure 4:**
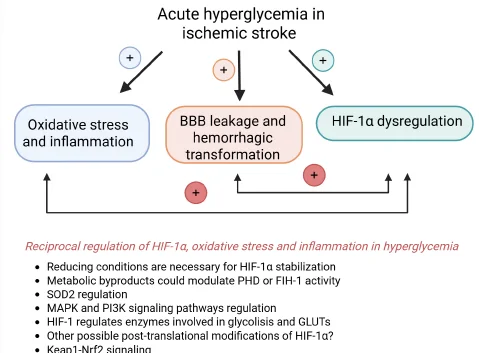
Hyperglycemia-HIF-1α imbalance-mediated stroke injury pathways. Created with BioRender.com. BBB: Blood–brain barrier; FIH-1: asparaginyl hydroxylase; GLUTs: glucose transporters; HIF-1α: hypoxia-inducible factor 1α; MAPK: mitogen-activated protein kinase; PHD: prolyl hydroxylase; PI3K: phosphoinositide 3-kinase; SOD2: superoxide dismutase 2.

HIF-1α regulates the expression of almost all enzymes involved in glycolysis, as well as GLUT1 and GLUT3, which mediate cellular glucose uptake. In turn, glucose availability, glucose uptake, and glycolysis influence the stability and activation of HIF-1α. The role of ROS in HIF regulation remains controversial, as ROS can up- or downregulate HIF depending on ischemic conditions (Xiao et al., 2013; Catrina, 2014). However, a reducing environment is necessary to stabilize HIF-1α in neurons exposed to hypoxia, and this stabilization is influenced by glucose concentration (Guo et al., 2008). A recent review has highlighted the close association between hyperglycemia, oxidative stress, and inflammation, which are prominent features in several human metabolic dysfunctions and pathologies (**Figure 4**; González et al., 2023).

Preclinical analyses suggest that HIF-based therapeutics hold promise for the treatment of inflammatory diseases. To fully realize the therapeutic potential of drugs targeting the HIF pathway, it is essential to understand the complex interactions among HIF, cellular metabolism, and immunity. A growing number of studies reveal that changes in cellular metabolism caused by hypoxia significantly influence immune cell activity. While the traditional oxygen-sensitive HIF pathway is observed in many cell types, research from the past decade shows it may be regulated by other mechanisms as well (Kuschel et al., 2012). These factors allow for distinctive qualitative, quantitative, and temporal variations in the HIF response, depending on the specific cellular and environmental context.

**Figure 5:**
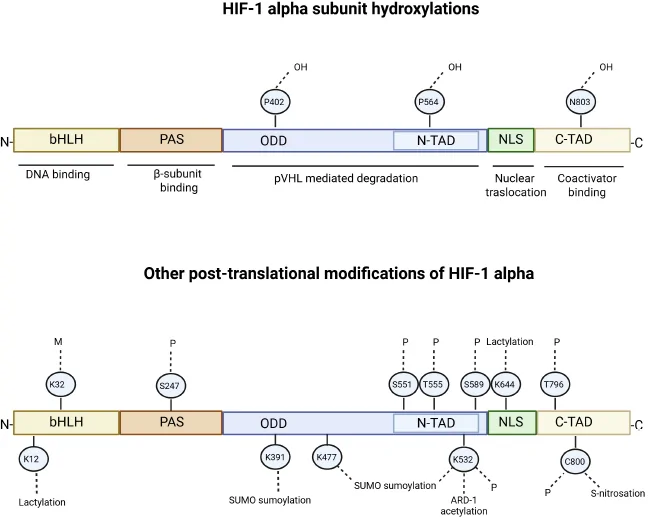
Domain structure of the human HIF-1α subunit. Key amino acid residues susceptible to hydroxylation under normoxic conditions include Pro402, Pro564, and Asn803. Additional post-translational modifications — such as methylation (M), phosphorylation (P), sumoylation, acetylation, S-nitrosation, and lactylation — occur at specific sites and influence the stability and transcriptional activity of HIF-1α. Created with BioRender.com. bHLH: Basic helix-loop-helix domain; C-TAD: C-terminal transactivation domain; HIF-1α: hypoxia-inducible factor 1α; NLS: nuclear localization signal; N-TAD: N-terminal trans-activation domain; ODD: oxygen-dependent degradation domain; PAS: protein/protein interaction and dimerization domain; pVHL: Von Hippel–Lindau tumor suppressor protein.

Post-translational modifications of HIF-1α, such as SUMOylation, phosphorylation, acetylation, S-nitrosylation, and methylation, have been extensively documented and influence the stability and activity of the HIF-α subunits (Mazure et al., 2004; Albanese et al., 2021). The post-translational modifications described are mainly based on studies conducted on human HIF-1 or in human cell models, with some references to animal models without distinguishing between species (**Figure 5**). A recent study identified lysine lactylation as a novel post-translational modification that regulates the stability and function of HIF-1α (Li et al., 2025). Mechanistically, lactylation at specific lysine residues — K644 in murine HIF-1α and K12 in human and porcine HIF-1α — impairs the ability of hydroxylated HIF-1α to engage with the VHL E3 ubiquitin ligase, leading to reduced polyubiquitination and degradation.

Other metabolic byproducts — such as ROS, hydrogen sulfide, iron, and metabolites from the tricarboxylic acid cycle (e.g., 2-oxoglutarate, succinate, and citrate) — regulate HIF-1α responses by modulating the activity of PHDs or FIH-1 (Taylor and Scholz, 2022). Although succinylation has not been explicitly reported as a post-translational modification of HIF-α, its recognized role in modulating protein function suggests it may affect HIF-α activity, especially under metabolic stress such as hypoxia and ischemia, where succinate accumulates. This is particularly relevant in acute hyperglycemia, where elevated succinate promotes protein succinylation. Since succinate inhibits PHDs that normally target HIF-α for degradation, succinylation of HIF-α lysine residues could further stabilize the protein by preventing its degradation, thereby enhancing the hypoxic response in this context (Selak et al., 2005).

In addition to this mechanism, an alternative pathway linking hyperglycemia with HIF-1α and ischemic damage involves the Kelch-like ECH-associated protein 1 (Keap1)/nuclear factor erythroid 2 p45 subunit-related factor 2 (Nrf2) system. Upon activation by harmful stimuli, Nrf2, a transcription factor, translocates to the nucleus where it promotes the expression of detoxifying enzymes such as glutathione peroxidase, glutathione S-transferase, and glucose-6-phosphate dehydrogenase (G6PDH), enhancing cellular defense against oxidative stress and potentially modulating the cellular response to ischemia (Takahashi, 2021). Under normal, non-stressed conditions, Nrf2 is bound to its partner protein Keap1, forming a heterodimer that undergoes continuous degradation through the ubiquitin–proteasome system. This process keeps Nrf2 activity low in a healthy, resting brain. However, ROS can modify cysteine residues on Keap1, triggering structural changes that release Nrf2. Once freed, Nrf2 translocates into the nucleus where it binds antioxidant response elements to activate genes related to energy metabolism, antioxidant defenses, and mitochondrial biogenesis. After hypoxia, the subsequent reoxygenation phase leads to a surge in ROS production, suggesting that agents enhancing the Keap1/Nrf2 pathway might provide neuroprotection (Takahashi, 2021). Activation of Nrf2/antioxidant response elements signaling has been shown to mitigate hyperglycemia-induced injury in podocytes (Zhang et al., 2018) and SH-SY5Y cell lines (Liu et al., 2019), as well as reduce oxidative stress-related ischemic injury in mouse models of MCAO (Jian et al., 2024). In contrast, in cases of severe cerebral injury or during extended periods after ischemic stroke, elevated ROS levels are observed alongside a decline in Nrf2 expression and reduced levels of its associated target genes. This pattern indicates that Nrf2 and its downstream effectors could be involved in the modulation of PHD activity and the activation process of HIF-1α (Oh et al., 2016; Cyran and Zhitkovich, 2022).

Beyond its role in hypoxia, HIF-1α induces the production of growth factors, such as IGF-2 and transforming growth factor-α. These growth factors bind to their respective receptors, initiating signal transduction pathways that promote cell proliferation and survival, while also stimulating HIF-1α expression. Furthermore, hypoxia and cytokines in certain cell types activate MAPK and PI3K signaling pathways, which enhance HIF-1 activity and contribute to cell proliferation and survival. Increased HIF-1 transcriptional activity at target genes, including those encoding IGF-2 and transforming growth factor-α, establishes autocrine signaling loops that play a critical role in cancer progression (Ke and Costa, 2006).

The use of molecular markers related to HIF-1α signaling as indicators of ischemic damage has been explored. For instance, Tuomisto et al. (2004) observed that the upregulation of HIF-1α, VEGF, VEGFR-2, TNF-α, and IGF-related signaling pathways functions as a set of molecular signatures associated with the diagnosis of critical ischemia in human skeletal muscle (Tuomisto et al., 2004). Similarly, Ke and Zhang (2013) reported that changes in cerebrospinal fluid concentrations of HIF-1α, VEGF, nerve growth factor, and brain-derived neurotrophic factor have been investigated as potential biomarkers for evaluating ischemic brain injury and cognitive dysfunction in patients with cerebral infarction. However, due to the complex and context-dependent nature of HIF-1α, relying on it as a biomarker presents significant risks and challenges (Ke and Zhang, 2013).

Conversely, research on drugs and molecules that modulate HIF activity or regulation has been carried out using a variety of animal models and *in vitro* systems. Amin et al. (2024) provide an extensive overview of compounds targeting HIF, highlighting three main categories of modulators. First, HIF-1α stabilizers, such as dimethyloxalylglycine, deferoxamine, and cobalt chloride, act by inhibiting enzymes responsible for HIF degradation, thereby promoting its accumulation and neuroprotective effects in ischemic conditions. Second, direct inhibitors such as acriflavine suppress HIF-1α activity when prolonged activation contributes to tissue damage, particularly in later stages of ischemia. Lastly, indirect or natural modulators, including salidroside, arginine, and glycine, regulate HIF-1α through related signaling pathways such as PI3K/Akt and NF-κB, fostering anti-inflammatory responses and neuronal protection (Amin et al., 2024). Davis et al. (2019) categorize HIF-1α regulators according to their mechanisms of action, which encompass agents that decrease Fe²⁺ availability via iron chelation, compounds that supply competing metal ions, and 2-oxoglutarate analogs that disrupt the regulatory pathways of HIF (Davis et al., 2019). Notably, the PHD inhibitor Roxadustat has shown promising neuroprotective effects in preclinical models of cerebral ischemia (Moreton et al., 2023; Madai et al., 2024). However, its use remains experimental, and further investigation is required to fully understand the spectrum of post-translational modifications that regulate HIF-1α.

**Figure 6:**
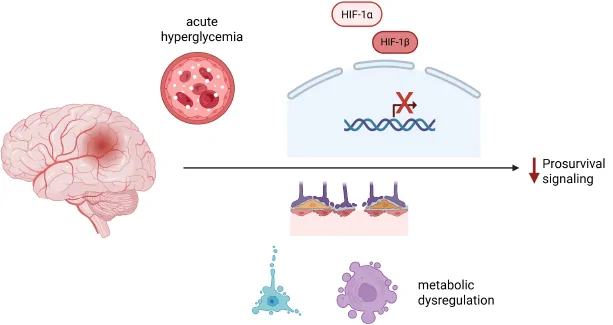
Potential role of hypoxia-inducible factor (HIF)-1α dysregulation in hyperglycemia-mediated injury during ischemic stroke. Acute hyperglycemia in ischemic stroke may modulate the activity of the transcription factor HIF-1 by affecting the stability of its alpha subunit. This dysregulation contributes to a disrupted metabolic response, resulting in diminished activation of cellular survival pathways during the early phases of ischemia. Created with BioRender.com.

To sum up, emerging evidence indicates that hyperglycemia may alter HIF-1α signaling during the early stages of cerebral ischemia, resulting in metabolic dysregulation and exacerbation of tissue damage (**Figure 6**). Since HIF is a transcription factor regulated by multiple factors beyond oxygen availability, its function is likely to be significantly affected by hyperglycemic conditions in the context of cerebral ischemia. Therefore, identifying the key enzymes that regulate the stability and activity of the HIF-1α subunit remains a critical priority for future research.

## Conclusions and Future Directions

The management of recurrent ischemic stroke is crucial for society due to its significant economic cost and the loss of healthy years of life. Clinical and preclinical trials demonstrate that hyperglycemia worsens the prognosis, likely due to the loss of the ability of tissue to adapt to hypoxic stimuli. Hyperglycemia appears to accelerate and exacerbate ischemic injury through increased oxidative stress and inflammation, leading to disruption of the BBB. Recent analyses on the variability of HIF protein stability have opened a potential therapeutic window for hypoxic pathologies. The study of acute hyperglycemia in ischemic stroke remains underexplored, but the role of HIF seems to be crucial. However, more data is needed on how HIF functions in transient ischemic models. In these models, compared to permanent ischemia, HIF appears to be beneficial. Additionally, further research is required to understand how HIF varies by cell type. The fact that many neuroprotective drugs are ineffective during the early hours after stroke underscores the need to further study into the role of other potential therapies.

In addition to the immediate harm to neurons caused by the harsh conditions of ischemia, much of the damage after stroke stems from secondary injury during reperfusion, which is inflammation-mediated. Interestingly, this initial, possibly detrimental response to acute damage also activates processes that promote recovery. Addressing factors that shorten the therapeutic time window for an excellent outcome could actually slow the progression of the penumbra’s evolution. HIF is a “multiple pathways” target and therefore a promising new approach for stroke treatment. It is interesting to consider that maybe the fact that hyperglycemia exacerbates injury is due to the lack of HIF-activated pathways and its protective role, which is lost beyond other mechanisms.

## Data Availability

*Not applicable.*
